# Small Molecule Screening with Laser Cytometry Can Be Used to Identify Pro-Survival Molecules in Human Embryonic Stem Cells

**DOI:** 10.1371/journal.pone.0054948

**Published:** 2013-01-29

**Authors:** Sean P. Sherman, April D. Pyle

**Affiliations:** 1 Molecular Biology Institute, University of California Los Angeles, Los Angeles, California, United States of America; 2 Department of Microbiology, Immunology, and Molecular Genetics, Eli and Edythe Broad Center of Regenerative Medicine and Stem Cell Research, Jonsson Comprehensive Cancer Center, University of California Los Angeles, Los Angeles, California, United States of America; Indian Institute of Toxicology Reserach, India

## Abstract

Differentiated cells from human embryonic stem cells (hESCs) provide an unlimited source of cells for use in regenerative medicine. The recent derivation of human induced pluripotent cells (hiPSCs) provides a potential supply of pluripotent cells that avoid immune rejection and could provide patient-tailored therapy. In addition, the use of pluripotent cells for drug screening could enable routine toxicity testing and evaluation of underlying disease mechanisms. However, prior to establishment of patient specific cells for cell therapy it is important to understand the basic regulation of cell fate decisions in hESCs. One critical issue that hinders the use of these cells is the fact that hESCs survive poorly upon dissociation, which limits genetic manipulation because of poor cloning efficiency of individual hESCs, and hampers production of large-scale culture of hESCs. To address the problems associated with poor growth in culture and our lack of understanding of what regulates hESC signaling, we successfully developed a screening platform that allows for large scale screening for small molecules that regulate survival. In this work we developed the first large scale platform for hESC screening using laser scanning cytometry and were able to validate this platform by identifying the pro-survival molecule HA-1077. These small molecules provide targets for both improving our basic understanding of hESC survival as well as a tool to improve our ability to expand and genetically manipulate hESCs for use in regenerative applications.

## Introduction

Human embryonic stem cells (hESCs) are derived from the developing blastocyst and have the potential to differentiate into all cell types in the body [1]. This pluripotent characteristic gives these cells enormous potential for both therapeutic uses and as a potent tool to study development *in vitro*. Development of applications for hESCs has been hampered by heterogeneity within stem cell cultures as well as variance in differentiation potential [2], [3]. One source of this variation is introduced by culture methodology: hESCs are typically passaged as clumps of cells due to poor survival as single cells [4]. This poor survival of individual hESCs increases the difficulty of manipulating these cells, which hampers the ability to clonally expand a cell line containing a desired modification from one integration event. One method of improving survival of hESCs is through the manipulation of proteins regulating apoptosis and the cell cycle. It has recently been shown that high levels of the protein Rem2 improve hESC survival by regulating Cyclin D1 and p53 to control the cell cycle and apoptosis of hESCs [5]. In addition, expression of anti-apoptotic proteins such as Bcl2 [6] or Bcl-XL [7] has been shown to improve the survival of single hESCs without altering their self-renewal or differentiation potential, however overexpression of oncogenes as a method of improving hESC survival would limit the therapeutic potential of the modified hESCs. A more transient method of improving hESC survival is through addition of growth factors or other small molecules to the culture medium [4], [8], [9]. It is now known that small molecule-mediated inhibition of Rho-associated protein kinase (ROCK) improves the survival of dissociated single hESCs [10], a result we confirmed using a novel high content screening assay [11]. ROCK inhibitors are also used to promote survival of hESCs during recovery from cryopreservation [12], [13], [14]. However, even treatment with ROCK inhibitors only results in a modest improvement in clonal efficiency of dissociated hESCs [15].

In our previous work, we identified a ROCK inhibitor, HA-1077, as promoting the survival of dissociated hESCs using a high content screening assay to test a small set of approximately 1600 compounds [11]. Other groups have also performed high content screening (HCS) assays using hESCs and have consistently identified multiple small molecules that promote hESC survival by inhibiting ROCK [15], [16], [17]. These screens, and our initial high content screen, all focused on small compound libraries or libraries composed primarily of characterized compounds and drugs with known targets. While screening well-characterized compounds can be helpful in understanding the mechanism of hits obtained, the commonly used libraries of well-characterized compounds, Biomol and Prestwick, have now been independently screened on hESCs by multiple labs, and all have identified only compounds that inhibit ROCK at various efficacies. In order to search for novel compounds that may regulate hESC survival by affecting non-ROCK pathways or to discover ROCK inhibitors with greater efficacies, we set out to screen the larger, more diverse libraries available at the UCLA Molecular Screening Shared Resource (MSSR).

In the work presented here we describe steps taken to optimize our previously published high content screening assay for use in screening large compound libraries. These optimizations include decreasing the length of the assay to more specifically target survival and changing the plate setup to a feeder-free culture system to reduce variation. In addition we have tested various DNA and viability dyes in order to replace the requirement for immunostaining in our previous screening assay, which is labor intensive and expensive. In total we evaluated three different readouts for survival using the Acumen laser cytometer, including live cell analysis of OCT4-EGFP using a reporter hESC line; use of the cell viability dye propidium iodide; and finally the cell viability dye Calcein AM. Using this optimized assay we screened ∼85,000 compounds for their effects on hESC survival. In order to distinguish surviving hESCs from background and non-specific signal, we developed a set of filtering parameters to use in quantifying surviving cells. The small molecule that produced the greatest improvement in hESC survival was confirmed to be a ROCK inhibitor, reinforcing the feasibility of this novel screening platform. Additional targets were found and although they were not as efficient at promoting survival as the ROCK inhibitor, they may prove useful in combination approaches to increase survival and increase our understanding of how survival is regulated in hESCs. In summary, in this work we have developed a novel screening platform using laser cytometry for use with hESCs without the need for fixation or extensive time or cost constraints. This optimized screening platform, which now enables large scale screening analysis of surviving hESCs after dissociation, could be used in further chemical screens, or in complementary DNA (cDNA) or RNA inhibtion (RNAi) based high throughput screens. Further this dataset provides a more comprehensive evaluation of chemical space for modifiers of survival in hESCs.

## Materials and Methods

### Cell Preparation for High Throughput Screening

hESCs (H1 OCT4-GFP [13] and H9 (WiCell) lines, passage 51–73) cultured under standard conditions [18] on mitomycin C-inactivated CF1 mouse embryonic fibroblasts (MEFs) were dissociated to single cells using 0.05% trypsin-EDTA (Life Technologies). After incubating 5 minutes in trypsin at 37°C, an equal volume of trypsin inhibitor (Life Technologies) was added. Cells were passed through a 40 µm mesh filter (BD Biosciences) and centrifuged at 1,000 rpm (200×*g*) for 5 minutes. After centrifugation cells were resuspended in 5 ml MEF conditioned medium (CM) and an aliquot was taken for live cell quantification using trypan blue exclusion. hESCs were then diluted to the desired concentration immediately prior to addition to screening plates.

### Screening Plate Preparation and Compound Addition

384 well screening plates (Greiner) were coated with hESC-qualified Matrigel (BD Biosciences) for one hour prior to addition of hESCs. Each aliquot of Matrigel was diluted in Dulbecco’s Modified Eagle Medium (DMEM)/F:12 (Life Technologies) as described in the product insert. 30 µl diluted Matrigel was added to each well of the screening plates using a Multidrop 384 equipped with a plate stacker (Thermo LabSystems). Plates were then incubated one hour at room temperature. After incubation, Matrigel was aspirated using an ELx 405 plate washer (Bio-Tek Instruments). 30 µl per well of CM was then added using a Multidrop 384. Screening compounds were then added using a Biomek FX (Beckman Coulter) in 0.5 µl DMSO for a final concentration of 10 µM. 20 µl per well of resuspended hESCs was added to prepared screening plates using a Multidrop 384 for a final assay volume of 50 µl. After addition of cells, screening plates were incubated 2 days at 37°C. In parallel, an aliquot of cells were taken and plated onto 6 well plates at each run to confirm viability of cells at the start of the run. Libraries screened include the Diversity, Targeted, Synergy, and Emerald sets from Asinex, the Chembridge DiverSet, Microsource and Enamine libraries, Biomol enzyme inhibitor and bioactive lipid libraries, and a collection of off patent FDA-approved drugs collected at the UCLA MSSR.

### Labeling of Surviving hESCs and Data Acquisition from Screening Plates

Three different methods were tested to quantify viable hESCs at the end of the screening assay. In all cases, data were acquired using an Acumen microplate cytometer (TTP Labtech). For quantification of GFP signal from the OCT4-GFP reporter, culture medium was aspirated and replaced with Dulbecco’s phosphate buffered saline (dPBS, Life Technologies), after which plates were immediately transferred to the Acumen system. For propidium iodide staining of cells, medium was aspirated and replaced with 50 µl per well 1 µg/ml propidium iodide (Life Technologies) in dPBS with 0.1% Nonidet P-40 (Fluka) to permeabilize cells. Cells were incubated 15 minutes at room temperature prior to data acquisition on the Acumen system. To use Calcein AM (eBioscience) to selectively label live cells, Calcein AM was diluted from 2 mM DMSO stock in dPBS to a concentration of 262.5 nM immediately prior to use. 20 µl per well of this solution was added to screening plates without removing culture medium. Plates were allowed to incubate 30 minutes at room temperature before data acquisition was performed on the Acumen system. To improve throughput, screening plates were stacked in a Twister plate handler robot (Zymark) integrated with the Acumen instrument. Data acquisition and analysis was performed using the Acumen Explorer software (TTP Labtech).

### Screening Approach and Analysis

Primary screens were performed to identify small molecules that increased the number of surviving hESCs 2 days after plating. Data were normalized on a per plate basis and results for each compound tested were expressed as a Z score, representing the number of standard deviations from the population mean for each plate. Compounds were identified as hits based on a sufficiently high Z score (described in each assay) and retested in the primary screening assay. Compounds that were confirmed to improve hESC survival in the primary screening assay were then tested in a OCT4 immunostaining assay to confirm that the increased number of surviving cells remained pluripotent. Any compounds that were confirmed to improve hESC in this secondary assay would next need to be tested in tertiary assays to characterize effects of the test compound on hESC proliferation, self-renewal, and differentiation as well as the mechanism of action of the pro-survival compound.

### Alkaline Phosphatase Assay

To test the effects of selected compounds on hESC survival of H1 OCT4-GFP or H9 hESCs were dissociated and plated on Matrigel coated 6 well plates in conditioned medium at an equivalent density per cm^2^ to the 384 well screening assays. After 2 days in culture the cells were fixed in 4% formaldehyde (from paraformaldehyde, Electron Microscopy Sciences). Alkaline phosphatase positive pluripotent cells were then stained by incubation in 0.01% Naphthol AS-MX phosphate (Sigma) containing 1 mg/ml Fast Red TR Salt (Sigma) for 30 minutes.

### Immunofluorescence Analysis of hESCs

hESCs were washed with dPBS and fixed with 4% formaldehyde at room temperature for 30 minutes. hESCs were then washed with dPBS, permeabilized in.1% Triton and blocked in 10% goat serum. Primary antibody (OCT4 used at 1∶100 dilution, Santa Cruz Biotechnology) was added in 1% goat serum overnight at 4 degrees (384 well plates). hESCs were washed with dPBS and FITC-conjugated secondary antibody (Thermo Scientific Pierce, used at 1∶500) was added for 1–2 hours at room temperature in the dark. To visualize nuclei, Hoechst 33342 (Molecular Probes) was added (1∶1000) in dPBS.

## Results

### A Shorter Incubation Period can be used to Identify Improved Survival in Dissociated hESCs

**Figure 1:**
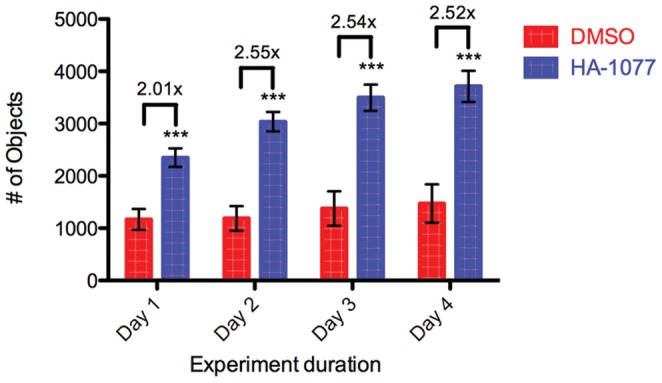
The greatest improvement in hESC survival due to treatment with HA-1077 is seen after two days. H1 OCT4-GFP hESCs were plated at 5000 cells/well on Matrigel in MEF-conditioned medium treated with 0.1% DMSO (red) or 10 µM HA-1077 (blue). Colored bars represent average number of GFP-positive objects identified ±SD. Values over bars represent fold increase in objects after treatment with HA-1077 relative to DMSO control. Statistical significance of HA-1077 treatment was determined using analysis of variance (ANOVA, ***: p<0.001).

Because hESCs undergo apoptosis rapidly following dissociation [19], [20], we sought to identify the shortest end point at which we could detect significant improvement in hESC survival due to small molecule treatment. In our previous work, we were able to identify small molecules improving hESC survival after 4 days in the screening assay [11], [18]. We observed that the magnitude of enhancement in survival after treatment with 10 µM HA-1077 increased from day 1 to day 2 post-plating, but remained the same from day 2 to day 4 post-plating (Figure 1). We also found that aspirating the culture medium, and presumably debris from apoptotic cells, increased the detectable fold-change in survival after treatment with HA-1077. In addition, we were able to plate dissociated hESCs in MEM-conditioned medium on Matrigel coated plates in place of the MEF co-culture system used previously.

### H1 OCT4-GFP hESCs Exhibit Dim Fluorescence in the High Content Assay Resulting in Additional Background and Nonspecific Fluorescence

**Figure 2:**
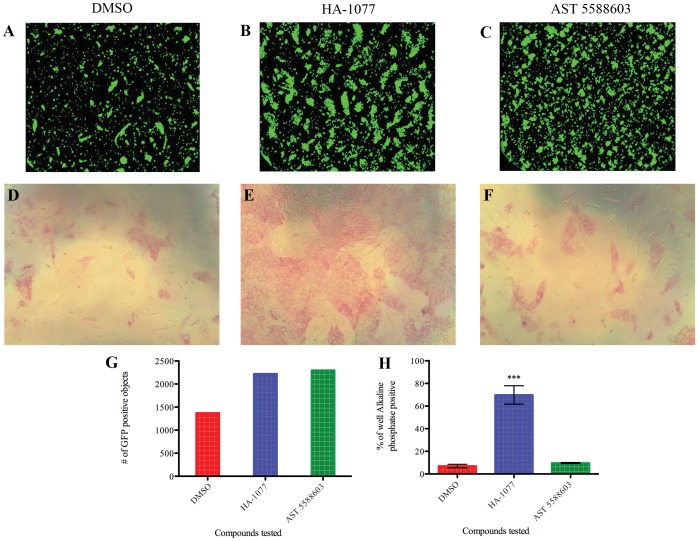
Small molecules identified in the OCT4-GFP screening assay were not confirmed in secondary assays. Three treatments are compared to assess changes in survival of dissociated hESCs: 0.1% DMSO (negative control; **A,D**), 10 µM HA-1077 (positive control; **B,E**), and 10 µM AST 5588603 (representative of small molecules identified in OCT4-GFP screening assay; **C,F**). **A-C:** Readout from the Acumen microplate cytometer. The wells treated with HA-1077 and AST 5588603 had similar numbers of GFP positive objects (quantified in **G**). **D-F:** Alkaline phosphatase staining of cells treated as described. Pluripotent cells are stained with a red color. Wells treated with HA-1077 (**E**) contain significantly more pluripotent cells than cells treated with DMSO or any other candidate compounds. **G:** Quantification of GFP signal detected in (**A-**C). **H:** Quantification of alkaline phosphatase staining in (**D-F**) by calculating what percent of the well area stained positive for alkaline phosphatase. Data shown as mean ±SD, ***: p<0.001.

After optimizing the high throughput assay with regard to culture conditions and duration we began to screen libraries of unknown compounds (See Table S1 for complete OCT4-GFP screen results). As compounds were identified in the Acumen screen as improving the number of GFP positive objects identified (Figure 2 A-C), we noted that the increased GFP signal did not correspond to a visible improvement in surviving cells after visual inspection under a light microscope. Wells with varying numbers of GFP-positive objects detected were examined manually under brightfield illumination and there was no noticeable correlation between GFP signal and the number of surviving hESCs. While positive control wells contained large highly compact hESC colonies, small molecule-treated wells displaying increased GFP-positive objects contained few surviving hESCs and large numbers of dead cells and aggregated debris. Four selected compounds that were identified as potential enhancers of hESC survival were retested in the 384-well high throughput format as well as with an alkaline phosphatase secondary assay to identify pluripotent cells. Of the four compounds tested out of 18860 compounds screened, only HA-1077 successfully improved survival of dissociated hESCs (Figure 2 D-F,H).

### Using a DNA Stain, Propidium Iodide, Presents an Alternative Readout for Detecting Survival of Dissociated hESCs in a High Throughput Assay

**Figure 3:**
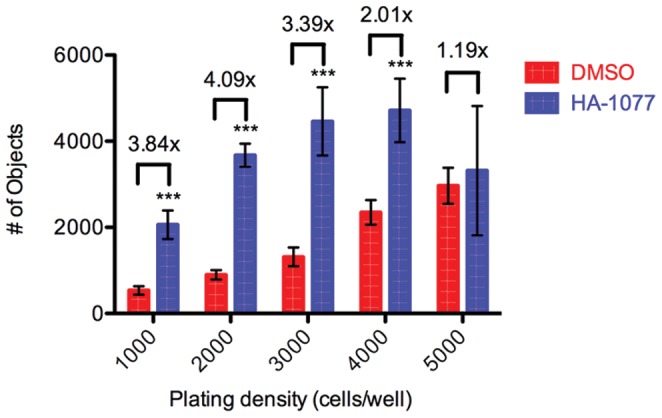
Lower hESC plating density results in greater observed changes in survival using propidium iodide. H1 OCT4-GFP hESCs were plated at the densities shown and treated with 10 µM HA-1077 (blue) or 0.1% DMSO (red). Colored bars represent average number of PI-positive objects identified ±SD. Values over bars represent fold increase in objects after treatment with HA-1077 relative to DMSO control. Statistical significance of HA-1077 treatment was determined using analysis of variance (ANOVA, ***: p<0.001).

We hypothesized that nonspecific GFP signal in the OCT4-GFP screening assay described above could be due to autofluorescence of cellular debris or the screening compounds tested. Because the Acumen microplate cytometer system does not require a focal plane for imaging, it has a higher sensitivity to background fluorescence that may not appear when using an image-based readout. Because the OCT4-GFP cells have dim GFP fluorescence even when OCT4 is highly expressed, even low nonspecific fluorescence could be mistaken for GFP signal. To overcome this nonspecific signal we tested the DNA stain propidium iodide in our hESC screening assay. Because propidium iodide is membrane impermeant, it was added to plates in a solution of 0.1% Nonidet P-40 detergent in dPBS. Used in a solution that would permeabilize cells, the propidium iodide was selected to stain DNA in hESCs that had survived and adhered to the tissue culture plate rather than serving as a direct test of viability. After noting that the number of objects stained with propidium iodide after 2 days was often similar between wells with and without ROCK inhibitor, we hypothesized that plating cells at a lower density would improve the detectable difference between positive (HA-1077 treated) and negative (DMSO vehicle) controls. We found that plating 2000 hESCs per well detected the largest increase in survival over control due to HA-1077 treatment (Figure 3). 17177 compounds that did not overlap with the OCT4-GFP screen were tested in the survival assay with propidium iodide readout (see Table S2 for complete results).

### Selective Labeling of Viable Cells Provides a More Specific Readout to Assess hESC Survival in High Throughput Assays

**Figure 4:**
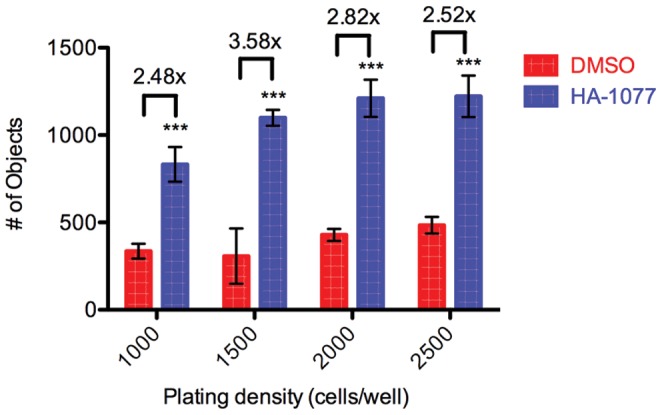
Altered hESC plating density improves the ability to detect changes in survival using Calcein AM. H1 OCT4-GFP hESCs were plated at the densities shown and treated with 10 µM HA-1077 (blue) or 0.1% DMSO (red) as a negative control. After 2 days viable cells were quantified. Colored bars represent average number of Calcein-stained objects identified ±SD. Values over bars represent fold increase in objects after treatment with HA-1077 relative to DMSO control. Statistical significance of HA-1077 treatment was determined using analysis of variance (ANOVA, ***: p<0.001).

While propidium iodide emits a more intense fluorescence than the EGFP in the OCT4-EGFP reporter cell line, it is unable to distinguish live and dead cells when used on permeabilized cells. Though the culture medium was replaced with propidium iodide in dPBS for staining, dead cells and debris that remained attached to the well surface were also stained with propidium iodide, leading to false positive results in high throughput assays screening 17177 compounds. To better distinguish surviving hESCs from dead cells and debris we used Calcein AM, a dye that fluoresces only after being taken up by viable cells and is retained within the cell once in its fluorescent state. As with the propidium iodide staining protocol, we tested multiple plating densities to determine the number of cells that resulted in the greatest improvement in positive control wells relative to untreated wells. We identified 1500 cells/well as the plating density that resulted in the greatest improvement in survival due to HA-1077 treatment (Figure 4). We then used a plating density of 1500 cells per well and a readout using Calcein AM viability dye after two days to assess hESC survival for screening the available small molecules in the UCLA MSSR.

### Evaluation of Primary Screening Data Identifies the Small Molecule HA-1077 as a Regulator of hESC Survival

**Figure 5:**
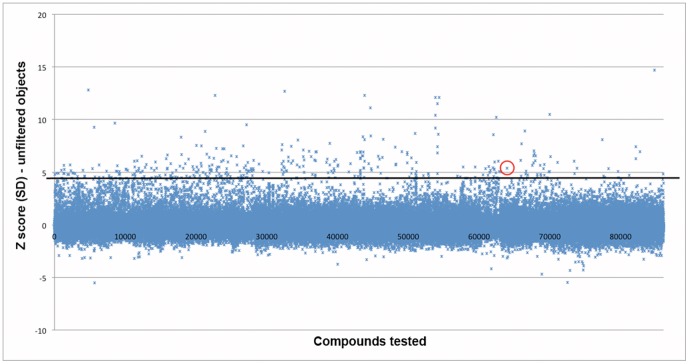
A Calcein based high throughput screen identifies potential regulators of hESC survival. Each blue point represents one of the 85586 small molecules tested. The black bar represents the cutoff (Z score ≥4.58) used to select a set of 320 compounds for follow up testing. The ROCK inhibitor HA-1077 is circled in red (Z score: 5.34).

Our complete screen tested 85586 small molecules with 85503 unique structures for their effects on hESC survival (Figure 5, Tables S3 and S4). For our initial analysis we chose to use the raw number of Calcein AM positive objects per well, to avoid introducing any bias due to selective filtering of results. We chose the 320 compounds with the highest Z score (Z ≥4.58) for repeat in the screening assay to confirm their effects on hESC survival. Included in this set of 320 was the ROCK inhibitor HA-1077.

**Figure 6:**
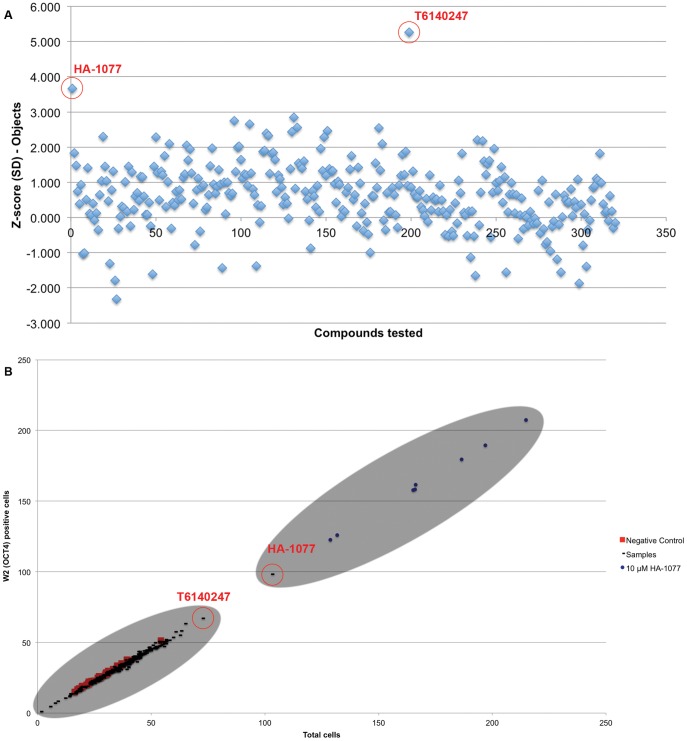
Secondary assays confirmed the pro-survival effects of the ROCK inhibitor HA-1077 on OCT4 positive hESCs. **A:** 320 candidate small molecules were rescreened in identical conditions to the primary assay. 2 small molecules circled in red, HA-1077 and T6140247, resulted in a significant (Z score ≥3 SD) improvement in viable Calcein positive objects. **B:** As a secondary assay, the 320 candidate molecules were screened at 1500 cells per well. After two days, cells were stained with the pluripotency marker OCT4 and counterstained with Hoechst 33342, in place of Calcein staining. The compounds identified in (**A**) are again circled. T6140247 is found clustered with other experimental samples (black) and negative control wells (red), while HA-1077 is found clustered with positive control wells treated with 10 µM HA-1077 (blue).

After repeating the primary Calcein screening assay with these 320 compounds, only two compounds produced an improvement in Calcein positive objects greater than three standard deviations from the plate mean (Figure 6A). To reconfirm the effects of these compounds on hESC survival we also tested the set of 320 compounds using our previous OCT4 immunostaining assay [11]. Only treatment with HA-1077 resulted in an improvement in the number of OCT4 positive cells (Figure 6B).

### Applying Constraints to the Object Identification Algorithm Improves the Ability to Distinguish Surviving hESCs from Background Signal

**Figure 7:**
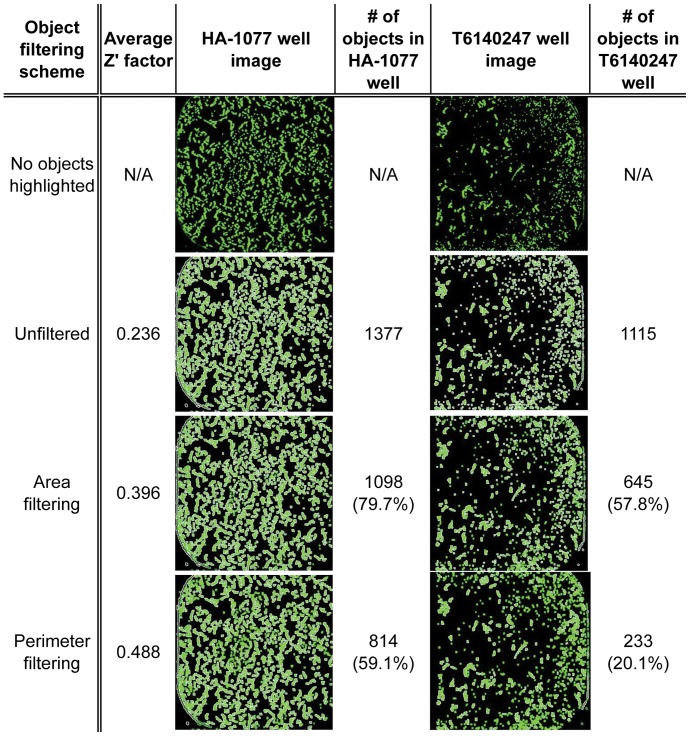
A perimeter-based object identification scheme results in the most robust detection of surviving hESCs. Three methods for quantifying Calcein staining of hESCs were compared: Unfiltered imposed no constraints on the object identification, while Area filtering requires fluorescent objects to have a perimeter ≥50 µm and area ≥125 µm^2^ to be counted and Perimeter filtering requires objects to have a perimeter ≥100 µm. Images compare the two compounds highlighted in Figure 4.6: HA-1077 (left) and T6140247 (right). For each category, objects identified are highlighted in white and quantified to the right of each image along with the percentage of the Unfiltered object count.

Using an unbiased object identification algorithm we identified ROCK as a key target for improving hESC survival. To identify additional potential hits improving hESC survival, we explored various filters that can be applied to specify when a fluorescent signal is classified as an object by the Acumen Explorer software. Parameters that were tested as potential filters include object dimensions (length & width), perimeter, and mean fluorescence intensity. We chose two object filter sets to characterize: one less stringent set that required fluorescent objects to have a perimeter ≥50 µm and area ≥125 µm^2^ to be counted and a more stringent set that required only an object perimeter ≥100 µm. These filtering strategies are referred to as area filtering and perimeter filtering respectively and can be used to distinguish pro-survival molecules such as the HA-1077 from molecules that were initially identified as hits but that did not display any pro-survival effects in follow-up testing such as T6140247 (Figure 7). While the two compounds register a similar number of objects without filtering, application of Area or Perimeter filtering more severely reduces the number of objects identified in the T6140247 well, consistent with the compound having no effect on the survival of dissociated hESCs. Because using the more stringent perimeter filter resulted in the highest average Z’ factor, representing the best separation of positive and negative controls, we used this filtering parameter to identify a second set of compounds with potential to improve hESC survival.

### Rescreening of Putative Hits from Revised Object Identification Strategy Confirms a ROCK Inhibitor as a Pro-survival Molecule in hESCs

**Figure 8:**
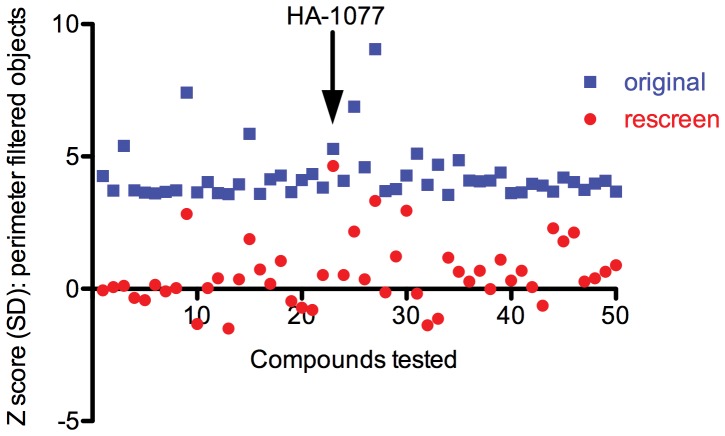
Rescreening of candidate molecules after perimeter based filtering identifies targets with pro-survival effects on hESCs. The top 50 compounds according to Z score of perimeter filtered objects, all with a Z’ factor >0.25, were repeated in the Calcein screening assay. Points represent the Z score, or number of standard deviations from the mean, of each sample in the original screening run (blue) or in the reconfirmation experiment (red).

After reanalyzing the 85586 compounds tested using our perimeter-filtered object identification strategy, we observed 392 compounds with a Z score ≥3 and a Z’ factor ≥0.25. A set of 50 compounds was identified by selecting the compounds with the largest Z scores (≥3.55) for perimeter-filtered objects after limiting the search to plates with a Z’ factor of at least 0.25 and compounds that had not been previously identified as hits in our initial evaluation of the primary data (Figure 6). These compounds were then rescreened in the primary Calcein based assay (Figure 8). All compounds but one fell below the Z score cutoff of 3 standard deviations from the mean that we set as a minimum requirement for positive hits. The only compound that produced a significant (greater than 3 SD) improvement in hESC survival was the ROCK inhibitor HA-1077.

## Discussion

In this work, we set out to optimize our high content screening assay to effectively screen larger libraries of small molecules than the ∼1600 tested in our first screening platform [11], [18]. Several aspects of the original high content assay setup and readouts were problematic in scaling up the assay, and are addressed in our optimized high throughput assay. Our first optimization was to switch from the previously published culture system of plating dissociated hESCs on MEFs to plating the cells on Matrigel coated plates in medium that had been conditioned overnight on MEFs. This optimization removed the presence of additional cell types in the screen, reducing the complexity of the screen. This was particularly helpful as we shifted the readout for the screen away from OCT4 positive cells to simply viable cells. A second change made to the assay was shortening the duration from 4 days to 2. We found that there was no difference in the ability of our assay to distinguish between positive and negative controls at 2, 3, or 4 days after plating (Figure 1) so we chose to use the shortest assay possible while still maintaining the dynamic range of the assay. While it is difficult to decouple survival and proliferation in an endpoint assay, using a shorter assay places more emphasis on initial survival of dissociated hESCs rather than proliferation after attachment. A shorter assay also reduces the likelihood of hESCs differentiating during the course of the assay. Characterization of hits in tertiary assays would be used to more closely examine changes in proliferation, self-renewal, and differentiation. Focusing our search on small molecules that improve the number of surviving hESCs in a shorter time frame improves the likelihood of finding small molecules affecting survival rather than self-renewal or proliferation.

Perhaps the largest change from the original high content assay is in the readout for surviving cells. While the original high content assay uses immunostaining for the pluripotency marker OCT4 as the readout for surviving hESCs, this readout is impractical for screening larger libraries due to both the reagent costs and the time and labor required to fix and stain hundreds of assay plates for immunofluorescence. Our first proposed readout was to measure OCT4 levels with a knock-in hESC line with EGFP driven by the OCT4 promoter [21]. However we found that the EGFP reporter was not sufficiently bright due to the fluorescent reporter itself, and as a result our screening assay was unable to sufficiently distinguish surviving hESCs from background signal from autofluorescing dead cells or fluorescent compounds (Figure 2). We next reasoned that a DNA-binding dye, propidium iodide, would limit our fluorescent signal to only cells (Figure 3). While the use of a non-specific DNA dye in place of an OCT4-dependent readout makes it impractical to verify the stemness of surviving cells, we were able to show that secondary assays could easily be used to assess whether novel small molecules were altering the self-renewal of the dissociated hESCs (Figures 2, 6). However, when testing the use of propidium iodide as a readout in our screening assay, we observed that hESCs that did not survive would often stick to the Matrigel coating the bottom of the well and would yield a positive signal mimicking viable cells. During assay development we were able to identify increased survival in groups of wells treated with positive control compounds, however in primary screening we observed well to well variability in the degree to which debris and dead cells were removed by washing, leading to many false positives where increased PI signal did not correlate with increased hESC survival. To address this we chose a dye that is only fluorescent when internalized by live cells. Calcein AM is the acetomethoxy derivative of the fluorescein-like dye Calcein. Calcein AM is nonfluorescent and cell-permeable until the acetomethoxy group is cleaved by intracellular esterases, at which point the dye becomes brightly fluorescent and is retained within the live cell. In the end we chose to use Calcein AM for our readout of surviving cells due to its bright signal and specificity to surviving cells. In the course of testing these readouts we found that plating dissociated hESCs at a lower density resulted in a larger dynamic range between positive and negative controls (Figure 4). Based on this analysis, we chose a plating density of 1500 cells per well for our complete screen.

After completing our screen of the available libraries at the UCLA MSSR (85586 compounds spanning 10 libraries) we then identified potential hits that appeared to improve the survival of dissociated hESCs. Although the software we used for data acquisition, Acumen Explorer, allows for limiting the fluorescent object identification according to various physical characteristics of the fluorescent objects, we chose to use the unfiltered object identification scheme to avoid potential biasing of our results. After normalizing results between plates, we chose the 320 compounds with the highest Z scores to assemble a cherry-picked plate to confirm the activity of these compounds in hESCs (Figure 5). To our surprise, the majority of the compounds we identified (318 of 320) had no effect on the number of Calcein positive objects in a repeat of the primary screening assay (Figure 6A). As an alternative assay we tested the cherry-picked 320 compounds with the same OCT4 staining readout as our previous work [11]. Although we found two compounds that gave a high signal in the Calcein assay (HA-1077 and T6140247) only one of them improved the survival of dissociated hESCs (Figure 6B).

In comparing the data acquired for HA-1077 and T6140247 we noticed that although both wells had a similar number of fluorescent objects, the objects in the T6140247 were mostly small punctate objects. In contrast, the objects in the HA-1077 well tended to be larger and better resembled growing hESCs. We then used these two compounds as test cases to develop a set of filtering parameters that excluded small points of fluorescence that were coming from a source other than surviving hESCs. While the number of objects detected decreased even in the HA-1077 (positive control) well, the decrease was much sharper in the well without increased numbers of hESCs (T6140247), suggesting that the correct object identification filters would help distinguish Calcein-stained hESCs from other sources of fluorescence (Figure 7). In addition to reducing the background signal from the small punctate non-cell objects, utilizing the perimeter filtering method improved the average Z’ factor of the assay from 0.236 to 0.488. The Z’ factor measures the distance between the positive and negative controls, with the most effective screening assays having a Z’ factor between 0.5 and 1.0 [22]. The assay Z’ factor using perimeter filtering is close to the Z’ factor of 0.5 observed in our previous screening assay, and suggests that the use of object identification filters will be important in optimizing future cell-based screens. Using the perimeter filtering method we selected the 50 compounds with the highest Z scores for cherry-picking to a new plate for retesting. After repeating our Calcein-based assay on these 50 compounds, we found that only the known ROCK inhibitor HA-1077 improved hESC survival by at least three standard deviations versus control treated wells (Figure 8). Interestingly, HA-1077 was also the only compound to result in a similar Z score in both the primary screen and the rescreening assay (5.29 and 4.63, respectively), while most compounds saw a decrease of 3–5 SDs when rescreened. However, we also found 6 compounds that resulted in a moderate improvement in hESC survival and could be investigated further in the future (Table S5). Additional evaluation of compounds with Z scores less than 3 may provide a method to identify small molecules with more modest effects on hESC survival that may be useful at a higher concentration or in combination with known ROCK inhibitors to promote hESC survival.

This work was initiated as a method to identify novel targets regulating the survival of dissociated hESCs beyond the known target ROCK. Although reports describing the mechanism by which ROCK is hyperactivated in hESCs after dissociation leading to apoptosis have identified other potential targets for regulating hESC survival [19], [20], other published screening assays have identified only small molecule inhibitors of ROCK as improving hESC survival [15], [16], [17]. As in this work we also identified the ROCK inhibitor, HA-1077, as the target with the greatest improvement in hESC survival, we are confident that our assay is capable of identifying small molecules that improve hESC survival to a degree similar to known pro-survival compounds. Further optimization of the assay could enlarge the distance between the positive and negative controls and could be used to search for novel targets that produce a more moderate improvement in hESC survival relative to that seen using known ROCK inhibitors.

The work demonstrates the feasibility of a high throughput, high content screen assessing survival of dissociated hESCs. Because ROCK inhibitors have been identified as the sole enhancers of hESC survival by multiple groups [15], [16], [17], it seems likely that any novel pathway promoting hESC survival has a more modest effect than that seen with ROCK inhibition in the current screening format. In the future, this assay could be performed in the presence of a ROCK inhibitor in order to screen for molecules that could be used in combination to further improve hESC survival in a fashion similar to our previous work in which we performed screens in the presence of ROCK inhibition as a method of identifying molecules that inhibit hESC survival [11].

In place of chemical libraries, cDNA or RNAi libraries could also be screened using a similar approach of quantifying viable cells after dissociation and replating. Screens using cDNA or RNAi libraries also have the benefit of well-defined and specific targets, compared to small molecules that frequently effect multiple targets at varying efficacies [23]. In summary, we have developed a high content screening assay to measure survival of dissociated hESCs that can be easily scaled up in order to test large libraries (85586 small molecules tested here). In this work, we confirmed the role of ROCK inhbition in improving survival and identified 6 more potential targets to follow up in future studies for use in combination with ROCK inhibitors. In addition, future applications for this optimized assay include continued screening of chemical libraries for novel regulators of hESC survival and screening of cDNA or RNAi libraries to shed light on additional players important for regulating survival.

## Supporting Information

Table S1 Normalized Z scores for OCT4-GFP based hESC survival screen.
(XLS)

Table S2 Normalized Z scores for propidium iodide based hESC survival screen.
(XLS)

Table S3 Normalized Z scores for Calcein AM based hESC survival screen (unfiltered results).
(XLS)

Table S4 Normalized Z scores for Calcein AM based hESC survival screen (perimeter filtered results).
(XLS)

Table S5 Normalized Z score results of rescreening in perimeter filtered screen.
(XLS)
